# Potential involvement of the 18 kDa translocator protein and reactive oxygen species in apoptosis of THP-1 macrophages induced by sonodynamic therapy

**DOI:** 10.1371/journal.pone.0196541

**Published:** 2018-05-10

**Authors:** Xin Sun, Shuyuan Guo, Wei Wang, Zhengyu Cao, Juhua Dan, Jiali Cheng, Wei Cao, Fang Tian, Wenwu Cao, Ye Tian

**Affiliations:** 1 Laboratory of Photo- and Sono-theranostic Technologies, Harbin Institute of Technology, Harbin, Heilongjiang, China; 2 Department of Cardiology, the First Affiliated Hospital, Cardiovascular Institute, Harbin Medical University, Harbin, Heilongjiang, China; 3 Department of Pathophysiology, the State-Province Key Laboratories of Biomedicine-Pharmaceutics of China, Key Laboratory of Cardiovascular Research, Ministry of Education, Harbin, Heilongjiang, China; 4 Materials Research Institute, The Pennsylvania State University, University Park, Pennsylvania, United States of America; Institute of Biochemistry and Biotechnology, TAIWAN

## Abstract

Sonodynamic therapy (SDT) with exogenous protoporphyrin IX (PpIX) or endogenous PpIX derived from 5-aminolevulinic acid (ALA) has been carried out to produce apoptotic effects on macrophages, indicating a potential treatment methodology for atherosclerosis. Our previous studies have found that mitochondria damage by reactive oxygen species (ROS) plays a major role in the SDT-induced apoptosis. This study aimed at investigating the potential involvement of the mitochondrial 18 kDa translocator protein (TSPO) and ROS in the pro-apoptotic effects of SDT on THP-1 macrophages. THP-1 macrophages were divided into control and SDT groups, and went through pretreatment of the specific TSPO ligand PK11195 and ROS scavengers N-Acetyl Cysteine (NAC), then compared with groups without pretreatment. Application of PK11195 reduced intracellular accumulation of endogenous PpIX. PK11195 and NAC reduced the generation of intracellular ROS and oxidation of cardiolipin induced by SDT, respectively. PK11195 and NAC also reduced SDT-induced mitochondrial membrane potential (ΔΨ_m_) loss, the translocation of cytochrome c and cell apoptosis. PpIX accumulation, ROS generation and cell apoptosis were also attenuated by siTSPO. Our findings indicate the pivotal role of TSPO and ROS in SDT-induced cardiolipin oxidation, ΔΨ_m_ collapse, cytochrome c translocation and apoptosis in THP-1 macrophages.

## Introduction

5-Aminolevulinic acid (ALA) is a natural precursor of protoporphyrin IX (PpIX) in the heme biosynthesis pathway. Over several intermediate enzyme steps outside the mitochondria, a ring system with four pyrrol-rings is synthesized from eight ALA molecules, yielding PpIX inside the mitochondria and finally heme via the addition of a ferrous ion to the center of the pyrrol-ring structure [1]. By adding ALA, the naturally occurring porphyrins PpIX may selectively accumulate in the mitochondria of cancer cells and inflammatory cells due to the limited capacity of porphobilinogen deaminase and ferrochelatase [2]. Such selectivity has been exploited in sonodynamic therapy (SDT) of tumor, a modality that involves the systemic administration of a tumor-localizing sensitizer and its subsequent activation by ultrasound through many mechanisms, such as sonoluminescence and sonochemistry, resulting primarily in reactive oxygen species (ROS)-induced apoptotic cell death [3, 4].

Macrophages participate in the lipid metabolism and inflammatory processes, and play a pivotal role in the progression and destabilization of atherosclerotic plaque [5, 6]. In a balloon-injured rabbit carotid artery model, we characterized the selective accumulation of ALA-PpIX in the atherosclerotic plaque. Moreover, ALA-PpIX in plaques was positively correlated with macrophage content, which provides a macrophage-selective treatment agent [7]. We also found that SDT with exogenous PpIX or endogenous ALA-PpIX induced apoptosis of macrophages *in vitro* [8] and *in vivo* [9], indicating a promising therapy for the treatment of atherosclerosis. However, the mechanism remains unclear.

The 18 kDa mitochondrial translocator protein (TSPO), formerly known as peripheral-type benzodiazepine receptor (PBR), can be found in glial cells of the brain and in cells of the peripheral tissues, including macrophages [10]. It is localized to outer mitochondrial membrane and physically associated with voltage dependent anion channel (VDAC) and adenine nucleotide translocator (ANT) that are the core components of the mitochondrial permeability transition pore (mPTP). It has been confirmed that TSPO has a high-affinity recognition site for porphyrins, particularly PpIX [11]. ROS, the main SDT-induced cytotoxic agent, can diffuse only approximately 0.01–0.02 μm in its lifetime [12]. The mPTP is thus expected to be one of the primary targets of ALA-mediated SDT. The damaged mPTP may trigger apoptotic processes by disruption of the mitochondrial transmembrane potential (ΔΨ_m_) and release of mitochondrial pro-apoptotic factors [13].

In this study, we used specific TSPO ligand PK11195 and siTSPO, as well as ROS scavengers N-Acetyl Cysteine (NAC) to investigate the role of TSPO and ROS in SDT treatment of THP-1 macrophages.

## Materials and methods

### Cell culture and experimental conditions

A human leukemic cell line, THP-1 cell (American Type Culture Collection, ATCC, Manassas, VA, USA), was cultured in RPMI 1640 medium containing 10% fetal bovine serum, 20 μg/ml penicillin and 20 μg/ml streptomycin at 37°C in a humidified atmosphere with 5% CO_2_. The cells were differentiated into macrophages by adding 100 ng/ml PMA for 72 hours. THP-1 macrophages were seeded in 96-well plates and incubated with ALA-PpIX, with or without PK11195 pretreatment. It has been confirmed that neither ALA administration nor ultrasound exposure alone shows significant effects on THP-1 macrophage apoptosis [8]. In this case, to characterize the roles of TSPO and ROS in the SDT-induced apoptotic process, THP-1 macrophages were cultured in 35-mm Petri dishes and received no treatment or SDT treatment, and with or without PK11195 and NAC.

### Drug treatment

ALA (1 mM) containing RPMI 1640 medium was added to the cultured THP-1 macrophages and incubated in the dark for 3 hours. The medium was then replaced by RPMI 1640 without ALA. For PK11195 and NAC pretreatment, the cells were seeded into the 35-mm Petri dishes. In the preliminary studies, the concentration of PK11195 (25 μM) was determined by examining the cytotoxicity of PK11195 (0–200 μM) on THP-1 macrophages and the effects of PK11195 (25 and 50 μM) on SDT-induced cell apoptosis (S1 Fig). The concentration of NAC (20 mM) has been determined in our previous study [8].

### Ultrasonic exposure system

After ALA incubation, the cells were exposed to the ultrasound, as previously described [8]. Briefly, the ultrasonic transducer, pulse generator and power amplifier used in this study were designed and assembled by the Harbin Institute of Technology (Harbin, China). The home-made ultrasonic transducer (diameter: 35 mm; resonance frequency: 1.0 MHz; duty factor: 10%; repetition frequency: 100 Hz) was placed in a water bath and 30 cm under the cells. The ultrasonic intensity used was 0.5 W/cm^2^, as measured by a hydrophone (Onda Corp., Sunnyvale, CA, USA).

### Fluorescence detection of endogenous ALA-PpIX

Intracellular PpIX was identified by a fluorescence microscope (Olympus, Tokyo, Japan). Fluorescence intensity of PpIX was measured by a fluorescence microplate reader (Titertek Fluoroscan II, Flow Laboratories, McLean, VA, USA) at 405 nm excitation and 635 nm emission wavelength.

### Reactive oxygen species detection

DCFH-DA was added to the medium of cells at a final concentration of 20 μM and incubated at 37°C for 30 minutes. The cells were observed using fluorescence microscopy before and at different time (0, 1, 2 and 3 hours) after SDT treatment. Then the cells were washed carefully with phosphate buffered solution (PBS). A total of 1×10^6^ cells were collected, resuspended in serum-free medium and measured using a fluorospectrophotometer (USB2000, Ocean Optics Inc., USA) at 488 nm excitation and 525 nm emission wavelengths.

### Cardiolipin oxidation analysis

Cardiolipin oxidation in mitochondria was determined with 10-N-Nonyl-Acridine Orange (NAO). Before and at different time (0, 1, 2 and 3 hours) after SDT treatment, macrophages were incubated with 10 μg/ml NAO for 15 minutes at 37°C in the dark and monitored by the fluorescence microscope. Then the cells were washed carefully with PBS twice. A total of 1×10^6^ cells were collected and measured by the fluorospectrophotometer at 480 nm excitation and 525 nm emission wavelengths.

### Mitochondrial membrane potential detection

The ΔΨm was assessed using fluorescent probe jc-1. The jc-1 is a dual emission potential-sensitive probe. Red fluorescence (Ex/Em = 425/590 nm) attributes to a potential-dependent aggregation in the mitochondria, and green fluorescence (Ex/Em = 490/530 nm), reflecting the monomeric form of jc-1, which appears in the mitochondria after ΔΨm loss. The emission spectra of jc-1 shift from green to red with increasing concentration (i.e. aggregation) in the mitochondria, thus, allows for a dual-color assessment of ΔΨm. Before and at different time (0, 1, 2 and 3 hours) after SDT treatment, macrophages were incubated with 10 mg/ml jc-1 for 20 minutes at 37°C in the dark. Cells from each sample were then analyzed by a FacsCalibur flow cytometer (Becton-Dickinson, USA).

### Cytosolic and mitochondrial cytochrome c measurement

Western blot analysis was performed to measure cytosolic and mitochondrial cytochrome c. Three hours after the SDT treatment, cells were collected. The mitochondrial and cytosolic fractions were obtained for Western blot analysis. Primary antibody was goat polyclonal anti-cytochrome c antibody (1:600). Secondary antibody was AP-IgG (1:500). The protein bands were quantified by a Bio-Rad ChemiDocTM EQ densitometer and Bio-Rad Quantity One software (Hercules, CA, USA). Actin was used as a loading control for the cytosolic fraction. HSP 60 was used as a loading control for the mitochondrial fraction.

### Cell apoptosis assay

Cell apoptosis was assessed by the Annexin V-FITC apoptosis kit according to the manufacturer’s instructions. Three hours after the treatments, the cells were incubated with 5 μl Annexin V and 5 μl PI for 10 minutes at room temperature in the dark. Cells from each sample were then analyzed by the flow cytometer. The data were analyzed using the CELLQuest software (Becton Dickinson, Franklin Lakes, NJ, USA). Cells in the lower-right quadrant (Annexin-V^+^/PI^-^) represent early apoptotic cells.

### RNA interference of TSPO

The small interference RNA of TSPO (siTSPO) was based on previously published paper: siTSPO 5’-CACUCAACUACUGCGUAUG-3’ [14]. SiTSPO and the scramble siRNA were synthesized by GenePharma (Shanghai, China) and were transfected into THP-1 derived macrophages with X-tremeGENE siRNA transfection reagent according to the routine process. Assessment of silencing efficiency was performed by western blot with the protein collected 48 hours after the transfection.

### Cell viability assay

During the experiment, the cells were seeded into the 35 mm Petri dishes and incubated with different concentrations of PK11195 (0–200 μM) for 24 hours. The survival rate of the cells was measured by MTT assay. Experiments were repeated three times independently.

### Isolation of murine peritoneal macrophages

Peritoneal macrophages were isolated from C57BL/6 mice (6–8 weeks old) 3 days after the intraperitoneal injection of 2 ml of 3% thioglycollate (Sigma-Aldrich). Five million peritoneal cells were plated in Petri dishes with RPMI 1640 medium containing 10% FBS and allowed to adhere for 4 hours. The purity of macrophages was identified by immunofluorescence staining for CD68.

### Mitochondrial membrane potential detection

Mitochondrial membrane potential was assessed using fluorescent probe jc-1. Macrophages were incubated with 10 mg/mL jc-1 for 20 minutes at 37°C in the dark. The fluorescence intensity was measured using a fluorospectrophotometer (Varian Australia Pty Ltd, Melbourne, Victoria, Australia) at 488 nm excitation and 530 nm (green) and 590 nm (red) emission wavelengths. Experiments were repeated three times independently.

### Statistical analysis

All data were reported as mean value ± standard deviation. A Shapiro-Wilk test was first used to test the normality of the data. One-way analysis of variance followed by Student-Newman-Keuls testing was used to determine the difference among the groups. Statistical evaluation was performed using Statistical Analysis System software (version 9.2, SAS institute, Cary, NC). Differences with *P* < 0.05 were considered statistically significant.

## Results

### PK11195 attenuated endogenous ALA-PpIX accumulation

**Fig 1:**
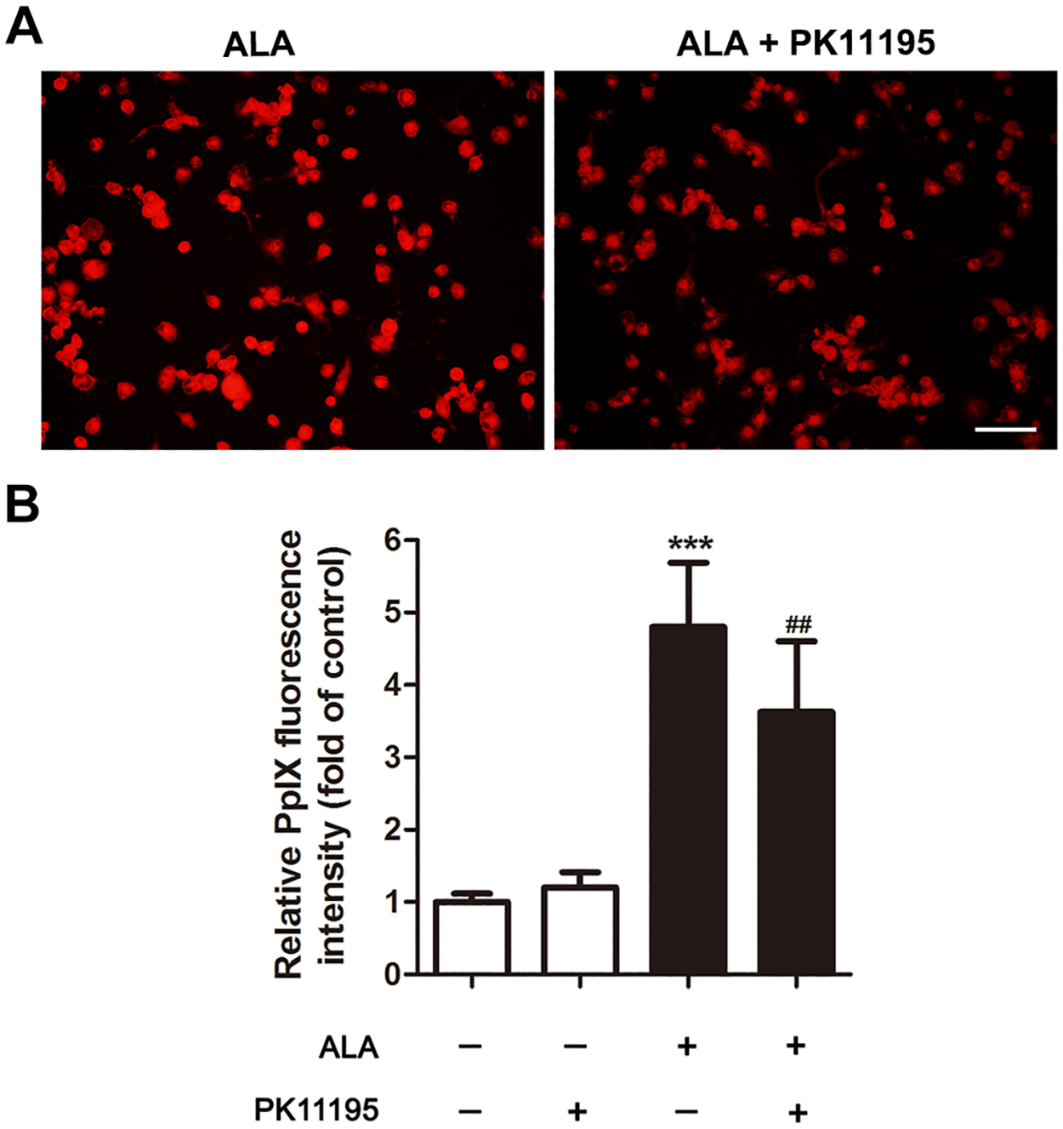
Effects of PK 11195 on endogenous ALA-PpIX accumulation. THP-1 macrophages were incubated for 3 hours with 5-aminolevulinic acid (ALA) with or without PK 11195. (A) Red fluorescence of intracellular PpIX was identified by fluorescence microscope. Scar bar: 0.1 mm. (B) Fluorescence intensity of PpIX was measured by fluorescence microplate reader. ***P < 0.001 compared to no treatment group, ^##^*P* < 0.01 compared to ALA treated group.

The fluorescence microscope detection showed that PpIX red fluorescence was observed in macrophages after incubation with ALA for 3 hours, which was decreased in cells pretreated with PK11195 (Fig 1A). The fluorescence intensity of PpIX was increased by 3.8 folds in cells incubation with ALA (*P* < 0.001), as compared with control. Pretreatment with PK11195 decreased endogenous ALA-PpIX fluorescence intensities by 24% (*P* < 0.01) (Fig 1B).

### Effects of PK 11195 and NAC on SDT-induced ROS generation

**Fig 2:**
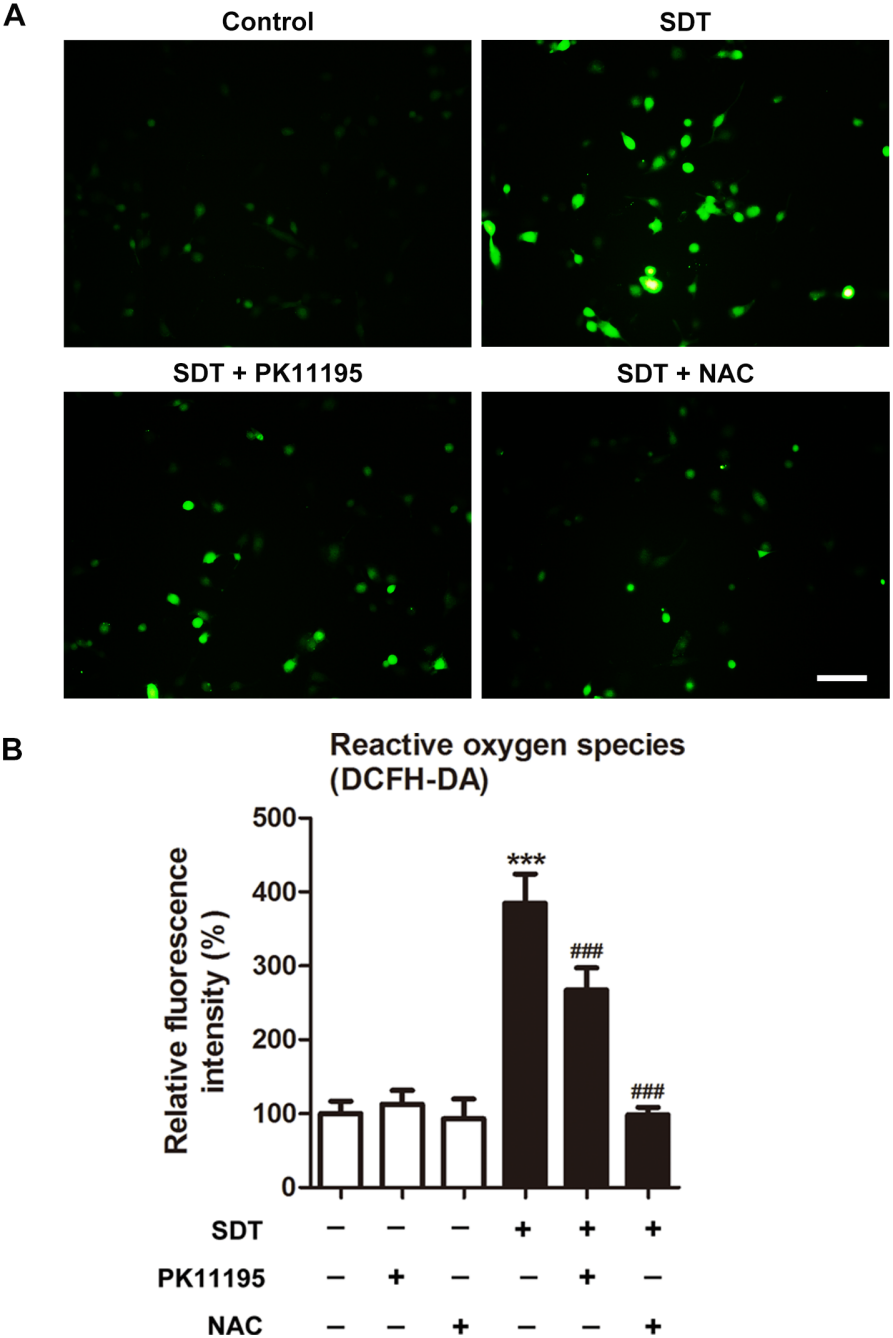
Effects of PK 11195 and NAC on SDT-induced ROS generation using the dye DCFH-DA. THP-1 macrophages were treated with 5-aminolevulinic acid mediated sonodynamic therapy (ALA-SDT), with or without pretreatment of PK 11195 or NAC. (A) Immediately after treatment, ROS production in THP-1 macrophages was observed under fluorescence microscope. The ALA-SDT treated cells showed increased green fluorescence level of ROS as compared with the untreated controls, and this effect was inhibited by adding PK 11195 or NAC before sonication. Scar bar: 0.1 mm. (B) Fluorescence intensity of ROS was measured using fluorospectrophotometer. ***P < 0.001 compared to no treatment group, ^###^*P* < 0.001 compared to ALA-SDT treated group.

Intracellular ROS generation was assessed by measuring the conversion of non-fluorescent DCFH-DA to fluorescent DCF. The green fluorescence of DCF was significantly increased at 0, 1, 2 and 3 hours after SDT (S2 Fig). The green fluorescence of DCF was present in few control cells and cells co-treated with SDT and NAC, but in a small portion of cells co-treated with SDT and PK11195, and most of the SDT-treated cells (Fig 2A). In accordance with this, the fluorescence intensity of DCF was increased (*P* < 0.001) in the SDT-treated macrophages, in comparison to the untreated controls. The generation of ROS by SDT was prevented by co-treatment with NAC and decreased by co-treatment with PK11195 (Fig 2B).

### Effects of PK 11195 and NAC on SDT-induced cardiolipin oxidation

**Fig 3:**
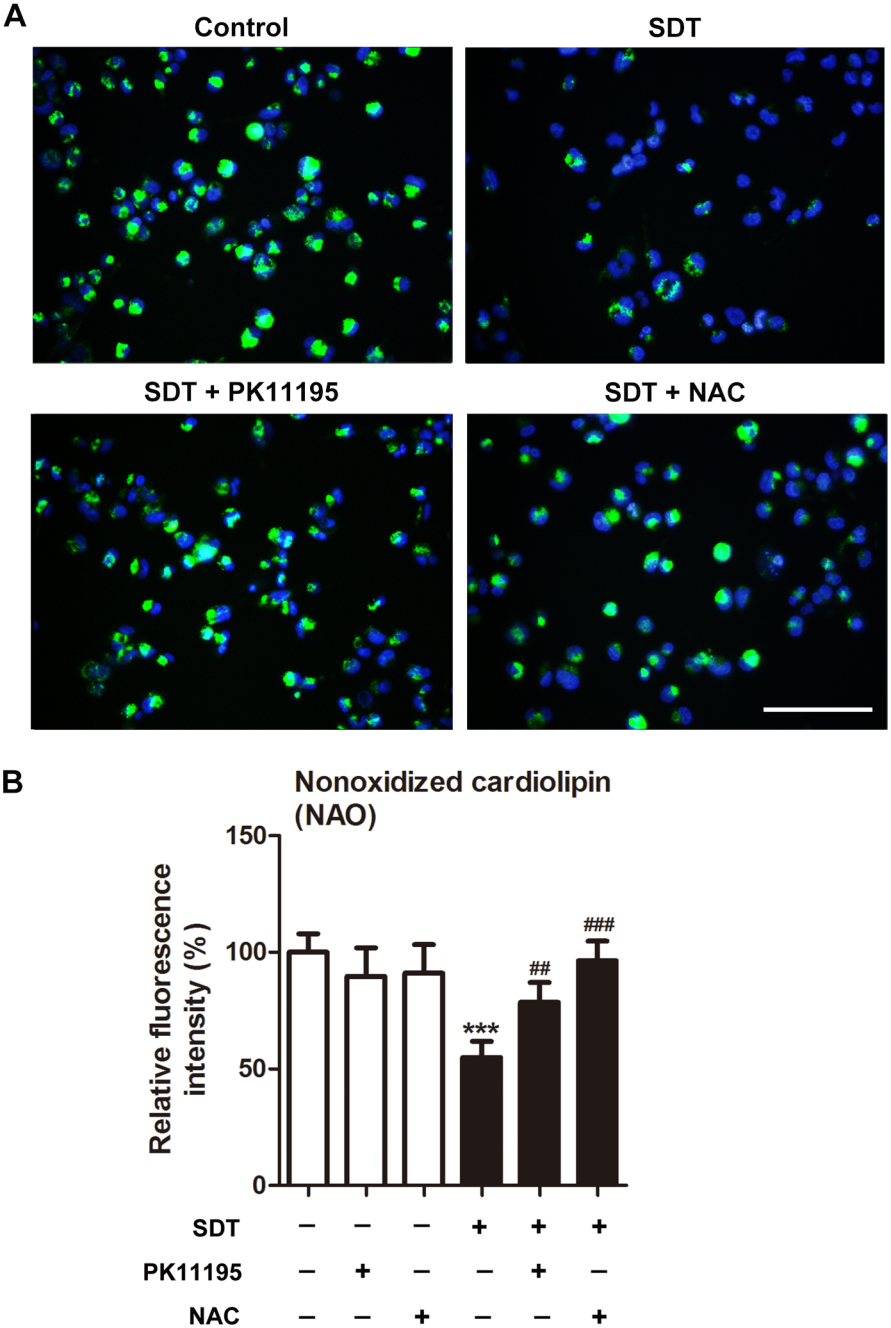
Effects of PK 11195 and NAC on SDT-induced cardiolipin oxidation. THP-1 macrophages were treated for 1 hours with 5-aminolevulinic acid mediated sonodynamic therapy (ALA-SDT), with or without pretreatment of PK 11195 or NAC, and cardiolipin oxidation was determined with 10-*N*-Nonyl-Acridine Orange (NAO). (A) Green NAO fluorescence was monitored by fluorescence microscope. Cell nuclei were stained with Hoechst. Scar bar: 0.1 mm. (B) Fluorescence intensity of NAO was measured using fluorospectrophotometer. ***P < 0.001 compared to no treatment group, ^##^*P* < 0.01 compared to ALA-SDT treated group, ^###^*P* < 0.001 compared to ALA-SDT treated group.

Cardiolipin oxidation in mitochondria was assessed by using NAO. The fluorescence microscope detection showed that NAO green fluorescence was observed within macrophages surrounding the nucleus. NAO green fluorescence was decreased at 0 hour after SDT, and further decreased at 1 hour (S2 Fig). In the control group, most cells showed a high level of NAO labeling. However, in the SDT group, cells showed a decreased level of NAO labeling as an indication of increased cardiolipin oxidation, which was prevented by co-treatment with PK11195 or NAC (Fig 3A). In accordance with this, the fluorescence intensity of NAO was reduced by 45% (*P* < 0.001) in the SDT-treated macrophages, in comparison to the untreated controls. Pretreatment with PK11195 and NAC, cells showed respectively 43% and 75% increase in the fluorescence intensity of NAO in comparison to SDT treatment alone (Fig 3B).

### Effects of PK 11195 and NAC on SDT-induced ΔΨ_m_ loss

**Fig 4:**
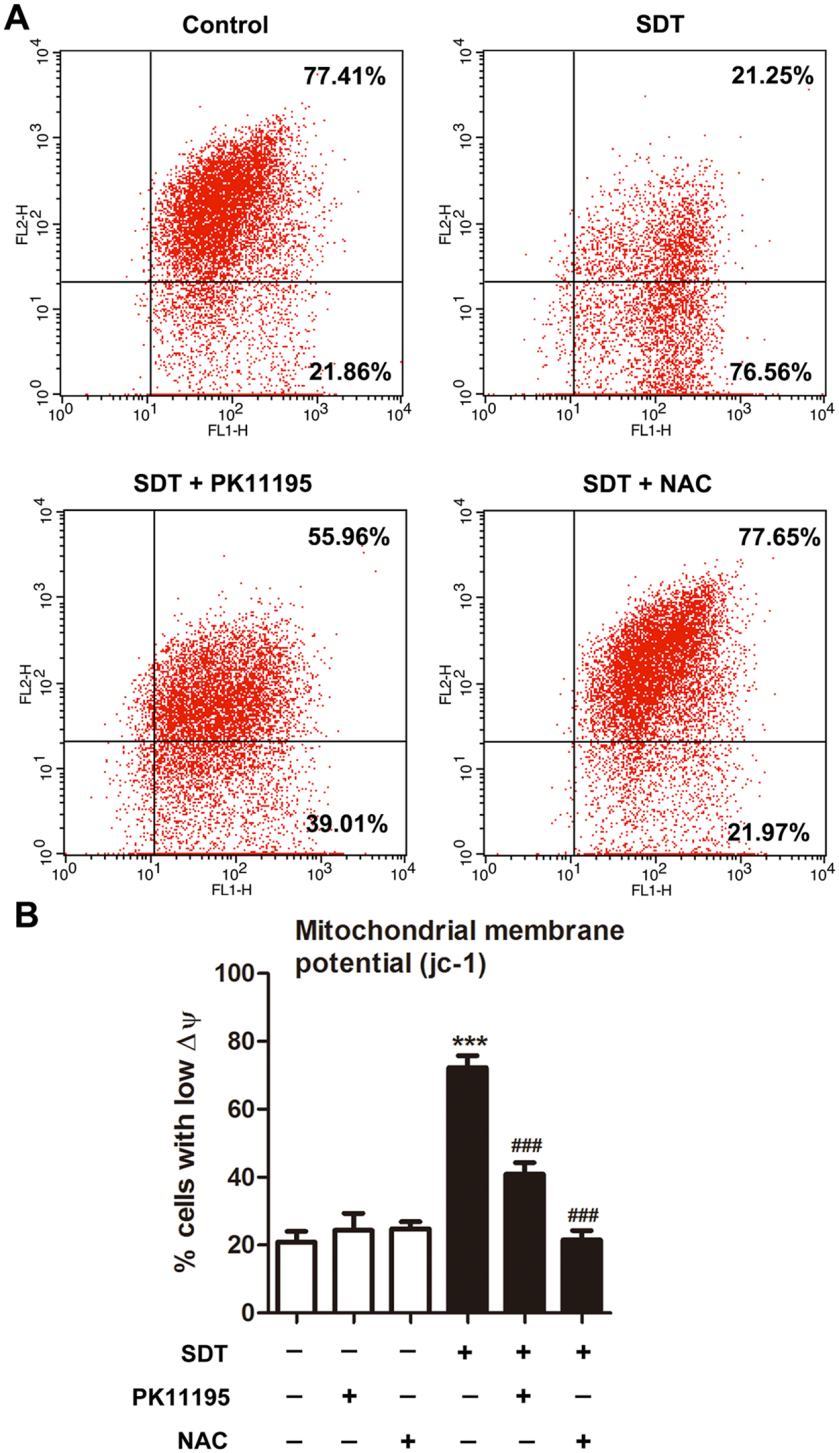
Effects of PK 11195 and NAC on SDT-induced ΔΨ_m_ loss, as determined with JC-1 by flow cytometer. THP-1 macrophages were treated for 1 hours with 5-aminolevulinic acid mediated sonodynamic therapy (ALA-SDT), with or without pretreatment of PK 11195 or NAC. (A) Cells with polarized mitochondria are found in the upper right quadrant, corresponding to high emission of fluorescence at both 590 nm (FL2-H, orange-red) and 527 nm (FL1-H, green), whereas cells with depolarized mitochondria are present in the lower right quadrant. (B) Quantifications of percentage of cells with low mitochondrial membrane potential (ΔΨ_m_) in the indicated groups. ***P < 0.001 compared to no treatment group, ^###^*P* < 0.001 compared to ALA-SDT treated group.

A ΔΨ_m_-sensitive dye, JC-1, was used to examine whether loss of ΔΨ_m_ is associated with SDT-induced apoptosis. Red fluorescence of JC-1 was decreased at 0 hour after SDT, and further decreased at 1 hour (S2 Fig). As shown in Fig 4A, cells with ΔΨ_m_ loss were present in the lower right quadrant. Three hours after the treatment, ΔΨm loss was seen in 20.91 ± 3.16% of the control cells, which was increased to 72.31 ± 3.52% in SDT-treated cells, and 40.87 ± 3.46% and 21.55 ± 2.72% in SDT-treated cells that co-treated with PK11195 and NAC respectively (Fig 4B).

### Effects of PK 11195 and NAC on SDT-induced cytochrome c translocation

**Fig 5:**
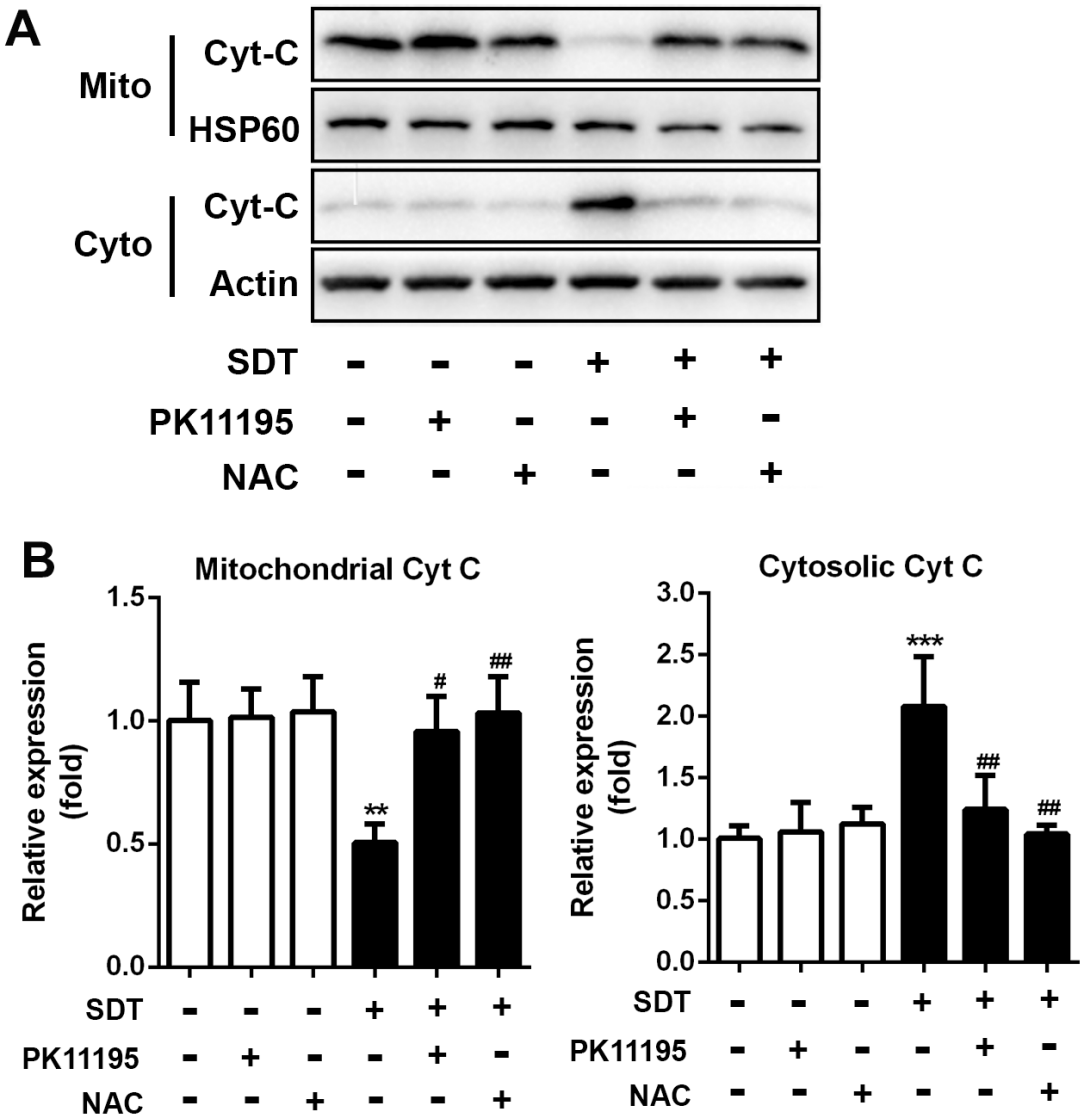
Effects of PK 11195 and NAC on SDT-induced cytochrome c translocation. THP-1 macrophages were treated for 3 hours with 5-aminolevulinic acid mediated sonodynamic therapy (ALA-SDT), with or without pretreatment of PK 11195 and NAC. (A) Cytosolic translocation of mitochondrial cytochrome c was examined by western blot of the cytosol and mitochondria. Actin and HSP60 were used as loading controls. (B) Quantitative representation of the Western blot. **P < 0.01, ***P < 0.001 compared to no treatment group, ^#^*P* < 0.05 compared to ALA-SDT treated group, ^##^*P* < 0.01 compared to ALA-SDT treated group.

The level of cytochrome c in the cytosol and mitochondria were determined by Western blotting. As shown in Fig 5A, the cytosolic cytochrome c level was low in the control cells. Low level of cytosolic cytochrome c was also present in SDT-treated cells pretreated with PK11195 and NAC, whereas high level of cytochrome c could be seen in the cytosol of SDT-treated cells. The mitochondrial cytochrome c level was high in the control cells. High level of mitochondrial cytochrome c was also present in SDT-treated cells pretreated with PK11195 and NAC, whereas low level of cytochrome c could be seen in the mitochondria of SDT-treated cells. Quantitative analysis revealed that the SDT-treated cells showed a 106% (*P* < 0.001) increase in cytosolic cytochrome c and a 50% (*P* < 0.01) decrease in mitochondrial cytochrome c as compared with the control cells, which was prevented by co-treatment with PK11195 and NAC (Fig 5B).

### Effects of PK 11195 and NAC a on SDT-induced macrophage apoptosis

**Fig 6:**
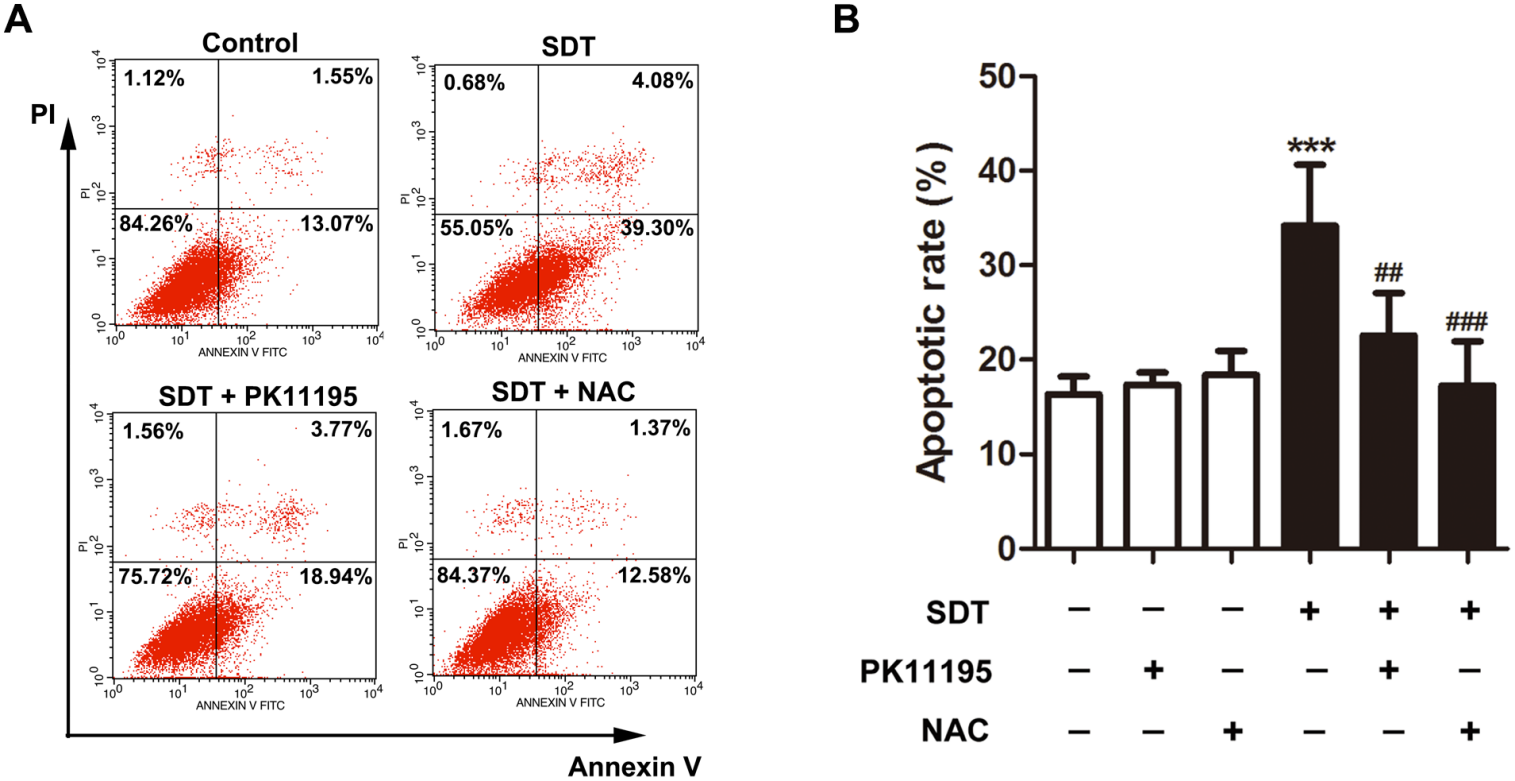
Effects of PK 11195 and NAC on SDT-induced macrophage apoptosis. THP-1 macrophages were treated for 3 hours with 5-aminolevulinic acid mediated sonodynamic therapy (ALA-SDT), with or without pretreatment of PK 11195 and NAC. Cell apoptosis and necrosis were assessed by flow cytometry with double staining of Annexin V and propidium iodide (PI). (A) Cells in the lower-right quadrant (Annexin-V^+^/PI^-^) represent early apoptotic cells. (B) Quantifications of early apoptosis rate in the indicated groups. ***P < 0.001 compared to no treatment group, ^##^*P* < 0.01 compared to ALA-SDT treated group, ^###^*P* < 0.001 compared to ALA-SDT treated group.

Cell apoptosis was measured using flow cytometry with double staining of Annexin V and PI. As shown in Fig 6A, early apoptosis was seen in 13% of the control cells. It was increased to 39% in the SDT-treated cells at 3 hours after the treatment. This increase was prevented in cells pretreated with PK11195 and NAC. Quantitative analysis showed that early apoptosis rate in the control group was 16.34 ± 1.88%. The SDT-treated cells displayed a 109% (34.21 ± 6.44%, *P* < 0.001) increase in early apoptosis rate in comparison to the untreated controls, whereas only 38% increase was observed in the cells pretreated with PK11195 (22.58 ± 4.50%) and 5% increase with NAC (17.25 ± 4.68%) (Fig 6B).

### Knockdown of TSPO attenuated PpIX accumulation, ROS generation and cell apoptosis by SDT

**Fig 7:**
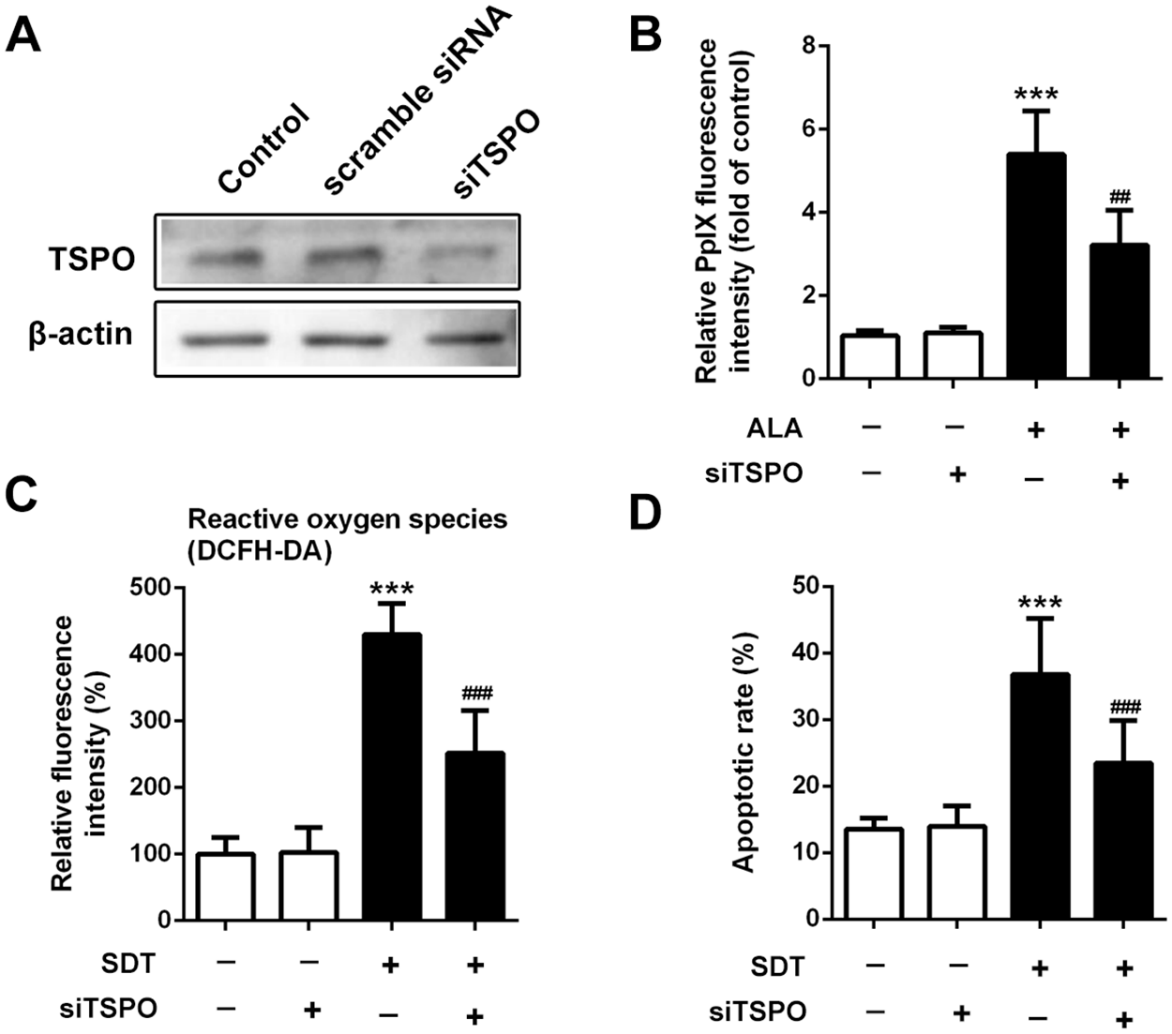
Effect of knockdown of TSPO on PpIX accumulation, SDT-induced ROS generation and cell apoptosis. (A) Knockdown of TSPO in THP-1 macrophages was examined by western blot. (B) Fluorescence intensity of PpIX was measured by fluorescence microplate reader. (C) Fluorescence intensity of ROS was measured using fluorospectrophotometer. (D) Quantifications of early apoptosis rate in the indicated groups. ***P < 0.001 compared to no treatment group, ^##^*P* < 0.01 compared to ALA-SDT treated group, ^###^*P* < 0.001 compared to ALA-SDT treated group.

The expression level of TSPO was significantly decreased in cells treated by siTSPO (Fig 7A). Treatment of siTSPO decreased intracellular ALA-PpIX fluorescence intensity by 40% (*P* < 0.001) (Fig 7B). The fluorescence intensity of DCF was decreased by 41% (*P* < 0.001) in the SDT-treated siTSPO macrophages, compared with non-siTSPO macrophages (Fig 7C). In addition, early apoptosis rate was decreased by 36% (*P* < 0.001) in the SDT-treated siTSPO macrophages, compared with non-siTSPO macrophages (Fig 7D).

## Discussion

This study demonstrates a TSPO-related pathway for macrophage apoptosis triggered by SDT. Previously, several other mechanisms have been reported. For example, SDT triggers DNA fragmentation and apoptosis in a syngeneic colon cancer model [15]. SDT damages mitochondria, activates pro-apoptotic factors Bax and cytochrome c in a human tongue squamous carcinoma SAS cell line [16]. Excessive intracellular ROS production followed by lipid peroxidation increase due to SDT has also been described in the SAS cells [17].

In this study, by applying TSPO ligand PK11195 and TSPO siRNA, we demonstrated that TSPO was involved in the process of SDT-induced macrophage apoptosis, including PpIX accumulation, ROS generation, cardiolipin oxidation, ΔΨm disruption and cytochrome c translocation. The similar effects were observed in the isolated of murine peritoneal macrophages (S3 Fig).

It has been reported that TSPO is involved in transport of porphyrins, like coproporphyrinogen III and PpIX across the mitochondrial membrane [18]. Several studies have also been published showing interactions of PpIX and TSPO in various cell models [19, 20]. This interaction was suggested to mediate the action of porphyrin based photosensitization in photodynamic therapy (PDT) of tumors [21]. Furthermore, it was demonstrated that selectively increasing TSPO expression in tumor cells by low-level light treatment facilitated ALA-PDT-induced tumor cell death [22]. In the present study, pretreatment with PK11195 or siRNA, the accumulation of PpIX was partially attenuated in macrophages (Figs 1B and 7B), which was in accordance with the previous studies in various tumor cells [23].

The decrease of ROS generation in the SDT-treated cells in the presence of PK11195 was expected from the reduction of intracellular ALA-PpIX accumulation. Intracellular ROS generation was also decreased in the presence of NAC, indicating that SDT produced ROS in macrophages (Fig 2). In addition, generation of ROS by SDT within mitochondria was confirmed by assessing cardiolipin oxidation (Fig 3). Likewise, siTSPO decreased ROS generation by SDT (Fig 7C).

Because the targets of SDT are the sites where the ROS is produced, the mPTP associated with TSPO is among the targets for SDT. Previous studies have shown that opening of mPTP by ROS is involved in TSPO activation-induced apoptosis [24]. When the mPTP opens, the ΔΨm collapses as a consequence of the dissipation of the proton gradient generated in the mitochondrial intermembrane space, which is an early event of the apoptotic cascade [24]. In agreement with these data, we showed that the ΔΨm was disrupted as early as 1 hour after SDT. PK11195 and NAC attenuated this effect (Fig 4), suggesting the involvement of TSPO and ROS in the SDT-induced ΔΨm disruption.

Disruption of ΔΨm has been reported to result in swelling of the mitochondrial matrix, mechanical rupture of the outer membrane, and release of inter-membrane proteins, such as cytochrome c and apoptosis-inducing factor [25]. Once released into the cytosol, these mitochondrial proteins mediate either a caspase-dependent apoptotic pathway or translocate further into the nucleus to induce a caspase-independent apoptotic pathway [26]. In the present study, we found that cytochrome c in the cytosol of SDT-treated macrophages was significantly increased (Fig 5), suggesting the translocation of cytochrome c from the mitochondria. This is in accordance with the fact that ROS generated by SDT induced dissociation of cytochrome c from cardiolipins after oxidized. Other studies have also found that ROS-induced VDAC alterations induce mitochondrial membrane permeability selective for cytochrome c release [27]. Increase of VDAC pore size via phosphorylation by protein kinase A, can be a mechanism of allowing cytochrome c release [28]. Moreover, assemblage of VDAC molecules into groups of up to 20 or even larger aggregates, including hexagonal packing, may play a role in cytochrome c release [29].

After cytosolic translocation, cytochrome c works with Apaf-1 and procaspase-9 in the presence of dATP or ATP to initiate apoptotic process by activating the downstream effector caspase-3 [30]. This is in accordance with our finding that early apoptotic cells were increased after the SDT treatment (Fig 6). Furthermore, SDT-induced cytochrome c translocation and cell apoptosis was almost abolished by PK11195 and siTSPO, as well as NAC, indicating the pivotal role of TSPO and ROS.

**Fig 8:**
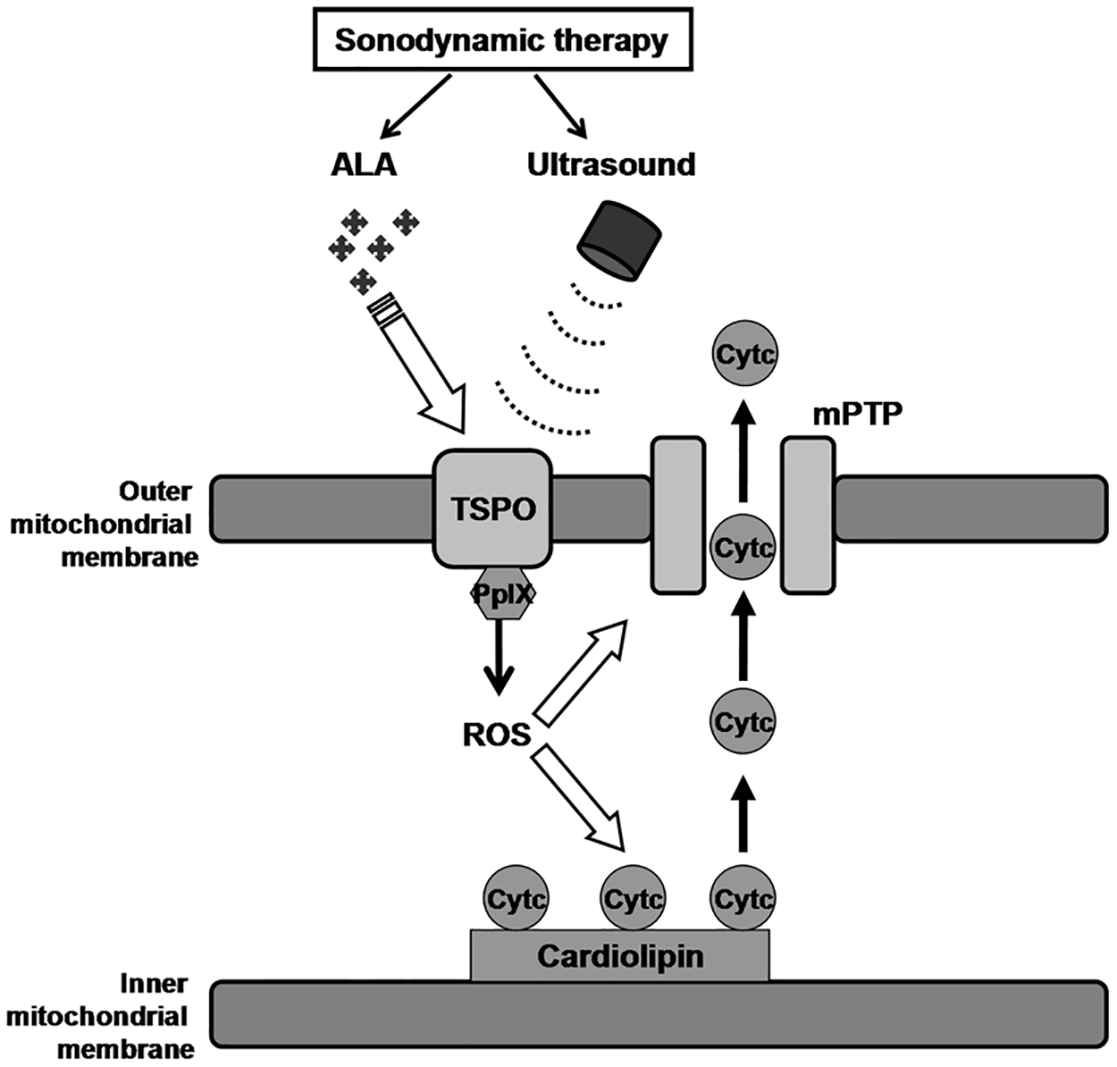
Schematic summary illustrating cytochrome c (Cytc) release due to reactive oxygen species (ROS) generation following activation of 5-aminolevulinic acid derived protoporphyrin IX (ALA-PpIX) binding to the 18 kDa mitochondrial translocator protein (TSPO) and subsequent opening of mitochondrial permeability transition pore (mPTP).

In conclusion, this study provides evidence that TSPO and ROS are involved in THP-1 macrophage apoptosis by SDT treatment. As shown in Fig 8, activation of ALA-PpIX binding to TSPO by ultrasound leads to ROS generation, resulting in the release of cytochrome c from oxidated cardiolipins at the inner mitochondrial membrane and the increase of permeability of the outer mitochondrial membrane, allowing cytochrome c translocation, which in turn induces macrophages apoptosis.

## Supporting information

S1 Fig Effects of PK11195 with different concentrations on cell viability and SDT-induced apoptosis in THP-1 macrophages. (A) Cytotoxicity of PK11195 on THP-1 macrophages with different concentrations was analyzed by MTT assay. (B) SDT-induced apoptosis was assessed by flow cytometry with double staining of Annexin V and propidium iodide (PI). ***P < 0.001 compared to no treatment group, ^###^P < 0.001 compared to SDT treated group.
(TIF)

S2 Fig Effects of SDT on ROS generation, cardiolipin oxidation and mitochondrial membrane potential loss in THP-1 macrophages. (A) Fluorescence intensity of ROS was measured by using fluorospectrophotometer with the staining of fluorescent probe DCFH-DA. (B) Cardiolipin oxidation was determined by using fluorospectrophotometer with the staining of NAO. (C) mitochondrial membrane potential was assessed by using fluorospectrophotometer with the staining of jc-1. **P < 0.01 compared to baseline, ***P < 0.001 compared to baseline.
(TIF)

S3 Fig Effects of PK11195 on ALA-PpIX accumulation, and ROS generation and cell apoptosis by SDT in isolated murine peritoneal macrophages. (A) Peritoneal macrophages were isolated from C57BL/6 mice and confirmed by immunofluorescent staining with CD68 antibodies. Scale bar represents 0.1 mm. (B) Fluorescence intensity of PpIX in the indicated groups detected by a fluorescence microplate reader. (C) Intracellular ROS generation in the indicated groups detected by fluorospectrophotometer with the staining of fluorescent probe DCFH-DA. (D) Quantifications of early apoptosis rate in the indicated groups measured by flow cytometry with double staining of Annexin V and PI. ***P < 0.001 compared to control group. ^##^P < 0.01, ^###^P < 0.001 compared to SDT group.
(TIF)
