# Supplementary figures and images for: Potential involvement of the 18 kDa translocator protein and reactive oxygen species in apoptosis of THP-1 macrophages induced by sonodynamic therapy
Source: PLoS One. 2018 May 10;13(5):e0196541. doi: 10.1371/journal.pone.0196541 (PMC5944937; doi:10.1371/journal.pone.0196541)

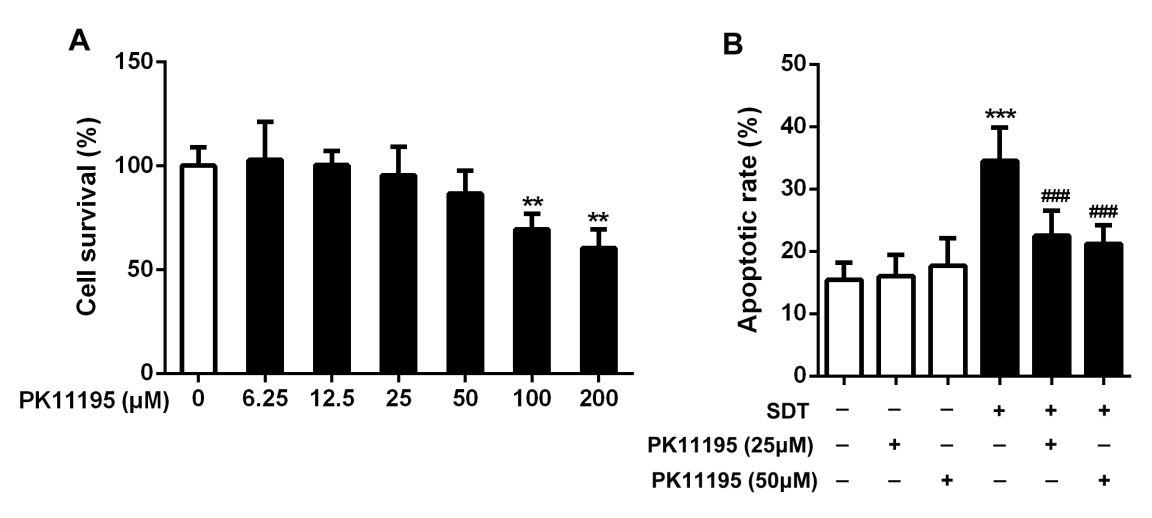

Supplement: S1 Fig — (A) Cytotoxicity of PK11195 on THP-1 macrophages with different concentrations was analyzed by MTT assay. (B) SDT-induced apoptosis was assessed by flow cytometry with double staining of Annexin V and propidium iodide (PI). ***P < 0.001 compared to no treatment group, ###P < 0.001 compared to SDT treated group. (TIF) [file pone.0196541.s001.tif]

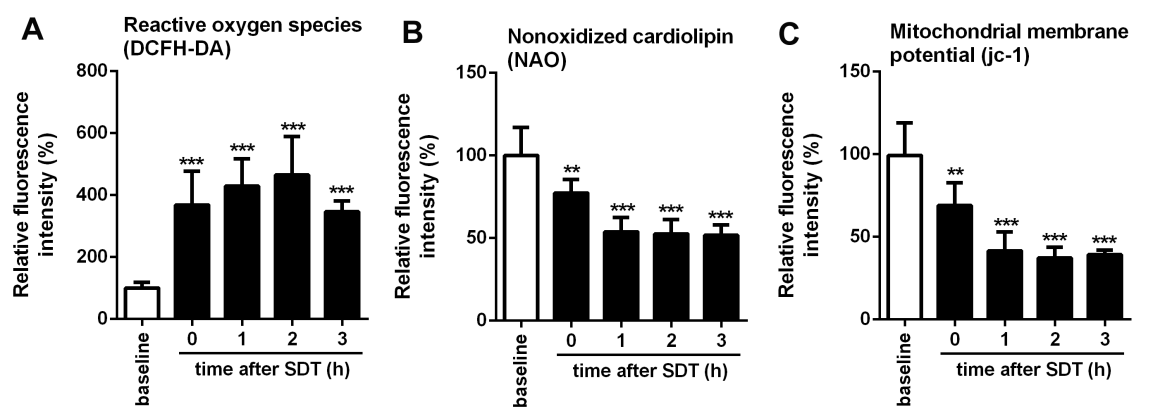

Supplement: S2 Fig — (A) Fluorescence intensity of ROS was measured by using fluorospectrophotometer with the staining of fluorescent probe DCFH-DA. (B) Cardiolipin oxidation was determined by using fluorospectrophotometer with the staining of NAO. (C) mitochondrial membrane potential was assessed by using fluorospectrophotometer with the staining of jc-1. **P < 0.01 compared to baseline, ***P < 0.001 compared to baseline. (TIF) [file pone.0196541.s002.tif]

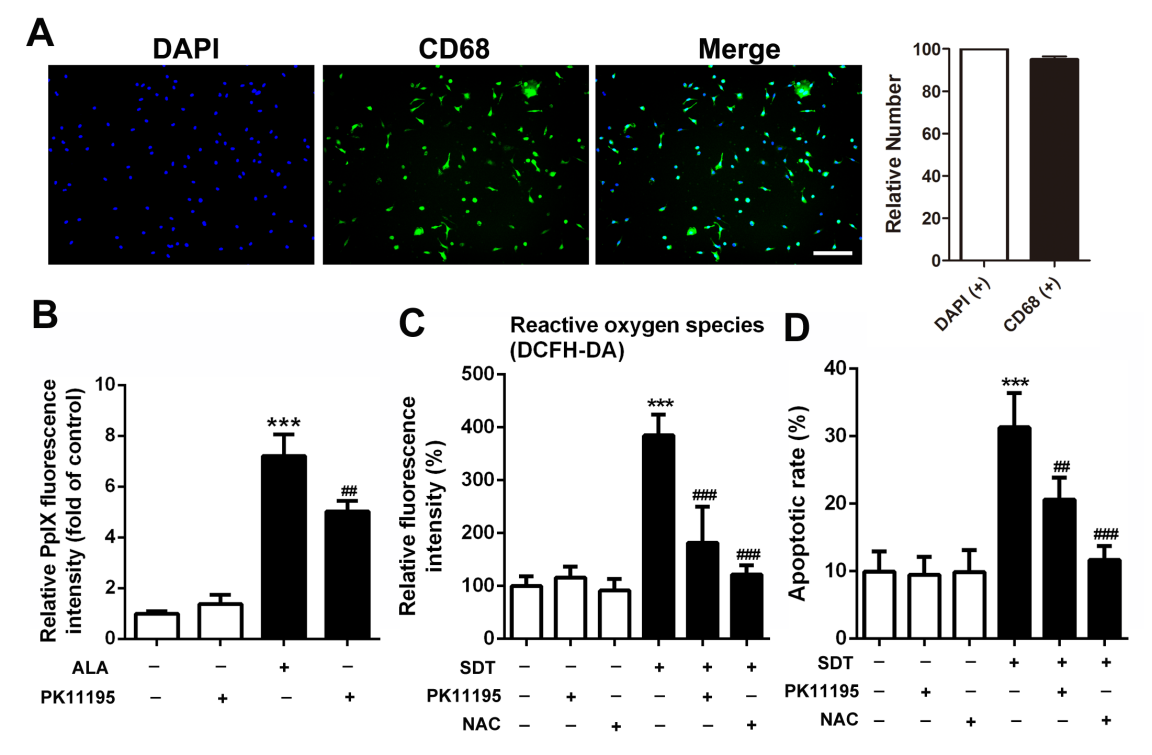

Supplement: S3 Fig — (A) Peritoneal macrophages were isolated from C57BL/6 mice and confirmed by immunofluorescent staining with CD68 antibodies. Scale bar represents 0.1 mm. (B) Fluorescence intensity of PpIX in the indicated groups detected by a fluorescence microplate reader. (C) Intracellular ROS generation in the indicated groups detected by fluorospectrophotometer with the staining of fluorescent probe DCFH-DA. (D) Quantifications of early apoptosis rate in the indicated groups measured by flow cytometry with double staining of Annexin V and PI. ***P < 0.001 compared to control group. ##P < 0.01, ###P < 0.001 compared to SDT group. (TIF) [file pone.0196541.s003.tif]
